# Fine-scale time-lapse analysis of the biphasic, dynamic behaviour of the two *Vibrio cholerae* chromosomes

**DOI:** 10.1111/j.1365-2958.2006.05175.x

**Published:** 2006-04-21

**Authors:** Aretha Fiebig, Kinneret Keren, Julie A Theriot

**Affiliations:** Department of Biochemistry, Stanford University School of Medicine, Beckman Center279 W. Campus Dr, Stanford, CA 95305, USA.

## Abstract

Using fluorescent repressor-operator systems in live cells, we investigated the dynamic behaviour of chromosomal origins in *Vibrio cholerae*, whose genome is divided between two chromosomes. We have developed a method of analysing fine-scale motion in the curved co-ordinate system of vibrioid bacteria. Using this method, we characterized two different modes of chromosome behaviour corresponding to periods between segregation events and periods of segregation. Between segregation events, the origin positions are not fixed but rather maintained within ellipsoidal caged domains, similar to eukaryotic interphase chromosome territories. These domains are approximately 0.4 µm wide and 0.6 µm long, reflecting greater restriction in the short axis of the cell. During segregation, movement is directionally biased, speed is comparable between origins, and cell growth can account for nearly 20% of the motion observed. Furthermore, the home domain of each origin is positioned by a different mechanism. Specifically, the *oriC*_*I*_ domain is maintained at a constant actual distance from the pole regardless of cell length, while the *oriC*_*II*_ domain is maintained at a constant relative position. Thus the actual position of *oriC*_*II*_ varies with cell length. While the gross behaviours of the two origins are distinct, their fine-scale dynamics are remarkably similar, indicating that both experience similar microenvironments.

## Introduction

For over 100 years, the dynamic behaviour of eukaryotic chromosomes within the nuclei of living cells and the dramatic events involved in chromosome segregation have been directly observed in the light microscope (Wilson, 1896). The organization and dynamics of bacterial chromosomes are more difficult to observe, and information on their behaviour has only recently emerged. Experiments in live cells have revealed that bacterial chromosomes also undergo a period of rapid segregation that may be analogous to eukaryotic anaphase (Glaser *et al*., 1997; Webb *et al*., 1998; Gordon *et al*., 2004; Viollier *et al*., 2004). Furthermore, at least in the case of *Caulobacter crescentus* and in slow-growing *Escherichia coli*, the bacterial chromosome is highly organized; chromosomal loci are ordered in a linear array throughout the cell that corresponds to their position on the chromosome (Niki *et al*., 2000; Viollier *et al*., 2004). In addition, multiple techniques have revealed that the bacterial chromosome is organized into a number of functional domains (for example Niki *et al*., 2000; Postow *et al*., 2004; Valens *et al*., 2004; Lesterlin *et al*., 2005; Stein *et al*., 2005). These findings have sparked widespread interest in bacterial chromosome dynamics, particularly during segregation. Less attention has been focused on chromosome dynamics between periods of segregation.

The positions of chromosomal loci in bacterial cells are highly stereotyped among individuals in a population and are correlated to their position in the genome. Specifically, the origin and termini regions typically reside at opposite ends of the nucleoid (Gordon *et al*., 1997; Webb *et al*., 1997; Niki and Hiraga, 1998; Lemon and Grossman, 2000; Li *et al*., 2002; Lau *et al*., 2003) and intervening loci occupy positions between the origin and terminus that correspond to their relative position on the chromosome (Teleman *et al*., 1998; Niki *et al*., 2000; Viollier *et al*., 2004). Similarly, segregation of the chromosome begins at the replication origin of the chromosome and proceeds sequentially through the chromosome to the terminus (Viollier *et al*., 2004; Bates and Kleckner, 2005; Fekete and Chattoraj, 2005; Wang *et al*., 2005).

Nevertheless, while there is order in the overall gross localization and segregation of bacterial chromosomes, individual origins exhibit positional variation (Gordon *et al*., 1997), and origins of fast-growing *E. coli* chromosomes are dynamic with apparently random motion (Elmore *et al*., 2005). The extent of mobility of individual positions on the chromosome is not well understood. In all population-based studies, where the location of a single chromosomal locus is determined in many individual bacteria, chromosomal loci have been found to occupy a broad range of positions. It is not clear to what extent the positional distributions reflect variations among individual bacteria or variations within individual bacteria that occur through time as a result of chromosomal movement. Importantly, the mobility of a chromosomal locus is under different constraints than a protein or other small molecule. A particular segment of DNA is covalently linked to a molecule many times longer than the cell itself and thus is compacted within the cell. Moreover, the DNA interacts with other cellular components via transcription, coupled translation and transertion. The active mechanisms operating during chromosome segregation in bacteria must separate the duplicated chromosomes in the context of these underlying constraints. We were therefore particularly interested in measuring the fine-scale mobility of bacterial chromosomal loci during the phases of the cell cycle between segregation events, and determining how the underlying mobility changes during active segregation.

We examined chromosome dynamics in *Vibrio cholerae* because it affords the opportunity to examine the behaviour of two distinct chromosomes in the same bacterial cell. Previous studies have indicated that both *V. cholerae* origins synchronously initiate replication once per cell cycle when grown in minimal media (Egan *et al*., 2004). Under these conditions, only one or two origins are expected for each chromosome per cell. In addition, the origins of both chromosomes have different steady-state localization patterns (Fogel and Waldor, 2005). Specifically, the origin of chromosome I occupies a near polar position and segregates asymmetrically from that position, while the origin of chromosome II localizes to the middle of the cell and segregates symmetrically. Moreover, chromosome segregation is not synchronous as it is in eukaryotic systems: the origin of chromosome I segregates early in the cell cycle and the origin of chromosome II segregates late in the cell cycle (Fogel and Waldor, 2005). Thus on a gross scale, the origins of the two *V. cholerae* chromosomes exhibit distinct behaviours. At the outset of this work, the fine-scale dynamic behaviour of the *V. cholerae* origins was not known. Here we quantitatively describe the dynamic behaviour of the origin region of both *V. cholerae* chromosomes during segregation and also between segregation events.

## Results

**Fig. 1:**
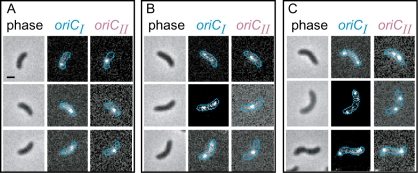
Localization patterns of *oriC*_*I*_ and *oriC*_*II*_. *oriC*_*I*_ exhibits a near-polar localization pattern, while *oriC*_*II*_ localizes to the mid-cell or the future mid-cell. *oriC*_*I*_ is visualized with LacI-CFP and *oriC*_*II*_ is visualized with TetR-YFP. A. Shorter cells have one focus for each origin. B. Mid-sized cells have two foci for *oriC*_*I*_ and a single focus for *oriC*_*II*_. C. Longer cells have two copies of each origin. Scale bar in the top left image (A) is 1 µm.

To monitor the behaviour of the *V. cholerae* chromosome origins in live cells, either *lacO* or *tetO* arrays were inserted near the origin of both chromosomes and visualized with LacI-CFP or TetR-YFP respectively (Lau *et al*., 2003). Simultaneous visualization of both origins reveals qualitatively distinct localization patterns for each origin (Fig. 1). These steady-state distribution patterns are the same in reciprocally marked strains (Figure S1) and confirm those described by Fogel and Waldor (2005) who characterized the positions of *lacO* and *tetO* arrays inserted at different origin-proximal sites. Together, these data indicate that neither the identity of the arrays nor the exact position of insertion affects the observed origin localization patterns.

**Fig. 2:**
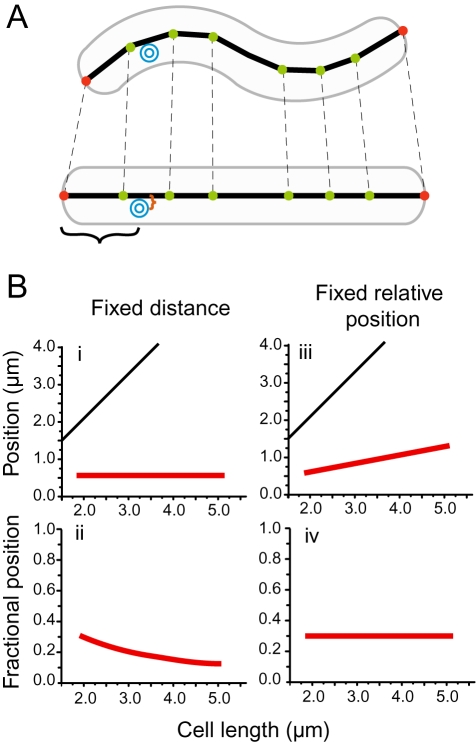
Frames of measurement. A. The positions of fluorescent foci (concentric blue circles) were measured based on objectively defined axes in these curved cells. The length of the cell is the sum of short linear segments (delimited by the green dots) along the centre of the bacterium. Red dots indicate the poles. The position of each focus was measured in terms of distance from the centreline (red bracket) and distance from the pole (black bracket). B. Expected line fitting analysis if origins were localized to fixed distances from the pole (i and ii) or fixed relative positions in the cell (iii and iv); see text for further details. In these examples, origins are a maintained at a distance of 0.5 µm from the pole (i and ii) or a relative position of 30% of the cell length (iii and iv).

For quantitative analyses of movement of subcellular features, it is important to consider the frame of reference for the measurements. Cell-based measurements are defined relative to a reference position in the cell such as a pole or the mid-cell. The non-uniform curvature of *V. cholerae* cells makes it difficult to directly translate fluorescent tag locations into cell-based co-ordinates. Young *V. cholerae* cells are curved to varying degrees and longer cells about to divide are often S-shaped. For quantitative position measurements throughout this study, we established an objective and general cell-based co-ordinate system corresponding to the length and width of the cell. The length of non-uniformly curved rods is measured as a sum of short linear segments along the centre of the cylindrical axis (Fig. 2). The two axes of the cell-based co-ordinate system correspond to positions along the centreline of the cell (length axis) and perpendicular distance from the centreline (width axis) (Fig. 2). In this way, we were able to measure origin locations using the length and width axes of the curved *Vibrio* cells across a population with varying shapes. We examined origin positions using both actual distances between the centre of the origin foci and a reference position in the cell such as a pole or the mid-cell, and fractional distances normalized by cell length. As shown in Fig. 2B, this analysis facilitated comparison of origin positions in large populations of cells as well as in individual cells over time (see below).

### Gross behaviour of *V. cholerae* origins

**Fig. 3:**
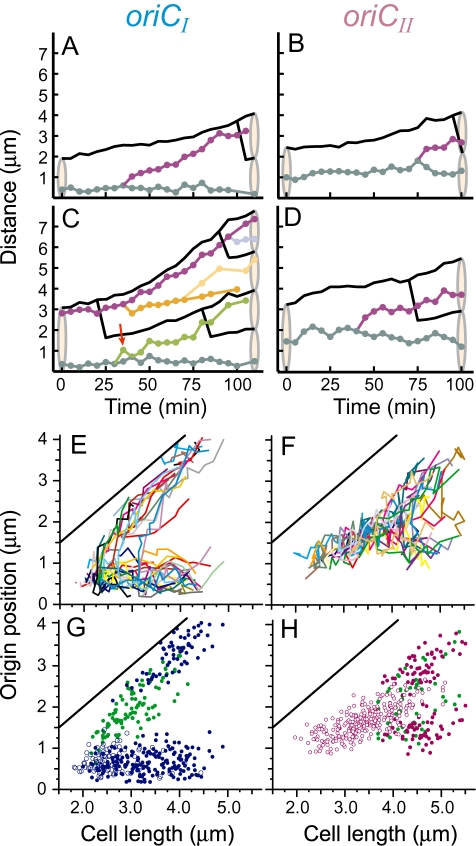
Time-lapse behaviour of both origins visualized with TetR-YFP over the course of 5 min intervals. Both *oriC*_*I*_ (A, C, E, G) and *oriC*_*II*_ (B, D, F, H) exhibit two behavioural phases corresponding (i) segregation when motion is directionally biased and (ii) periods between segregation events during which the position is not fixed but rather maintained in a ‘home’ domain (see text). Graphs A–D represent the tracks obtained from single cells over the course of about 100 min. The pink ovals represent the cell body at the beginning and end of the time-lapse. The black lines indicate cell length. The perpendicular branches in the black lines designate cell divisions. Arrow indicates an example of a ‘bounce’ at segregation, see text. To enable comparisons between cells, tracks of origin position were plotted versus cell length instead of time (E–H). When cells divided, tracks were subdivided so that each line in E or F represents a track in a single cell cycle. Thirty-two *oriC*_*I*_ were tracked in 30 cells and 28 *oriC*_*II*_ were tracked in 21 cells. Positions of origins in the home and segregating phases are indicated in G and H, where origin positions measured at each time point are plotted versus cell length without the lines connecting the tracks. A single origin in a cell is indicated by an open circle. Once segregated, origins are indicated by filled circles; those in the segregating phase are green, and those that remained at home, or have reached a new home are blue or red for *oriC*_*I*_ and *oriC*_*II*_ respectively.

For exploration of dynamic behaviour, we used time-lapse microscopy to track fluorescent foci corresponding to TetR-YFP bound to *tetO* arrays inserted near the origin regions of both *V. cholerae* chromosomes (∼13 kb counterclockwise from *oriC*_*I*_ and ∼12 kb counterclockwise from *oriC*_*II*_) as they moved over 5 min, 20 s and 1 or 2 s intervals. Tracks from the 5 min interval movies provide a general picture of the behaviour of each origin, examples of which are shown in Fig. 3A–D. To facilitate comparisons between cells, origin tracks from all cells were plotted as a function of cell length (Fig. 3E and F). These tracks from 5 min interval movies corroborate the large-scale behaviours previously described (Fogel and Waldor, 2005). First, each origin occupies a distinct region of the cell, with *oriC*_*I*_ near the poles and *oriC*_*II*_ near the mid-cell. Second, each origin exhibits a distinct segregation pattern; *oriC*_*I*_ segregates asymmetrically with one copy maintaining the original position, while *oriC*_*II*_ segregates symmetrically from the mid-cell. Third, separation of the tracks in Fig. 3E and F into individual points representing segregating and non-segregating points in the cell cycle (Fig. 3G and H) corroborates the sequential segregation of the two origins described by Fogel and Waldor (2005), with *oriC*_*I*_ segregating fairly early in the cell cycle when bacteria are ∼3 µm in length and *oriC*_*II*_ segregating later when bacteria are typically ∼4 µm or longer. These observations set the groundwork for further quantitative analyses.

Tracking *oriC*_*I*_ through cell divisions reveals that this origin remains near the old pole, as opposed to the new pole formed by the most recent cell division. Furthermore, once the *oriC*_*I*_ home position is established, it is maintained through subsequent cell divisions (Fig. 3A and C). Positioning of *oriC*_*II*_ followed a different pattern. Because the home for *oriC*_*II*_ is near the mid-cell, this position changes with each cell division (Fig. 3B and D). Tracking *oriC*_*II*_ through cell divisions reveals that the home position of *oriC*_*II*_ is biased towards the new pole. Of 18 origins observed through a cell division, 14 were closer to the pole recently formed by cell division.

In the initiation of segregation, we observed several cases where a second origin focus appears, then seems to disappear for one or more frames, and then reappears and persistently moves across the cell (for example see arrow in Fig. 3C). This was observed in three of 18 *oriC*_*I*_ segregations and three of 23 *oriC*_*II*_ segregations. This behaviour was not observed at other times in the cell cycle. While it is possible that this observation reflects some dynamic behaviour of the integrated arrays, we interpreted this to indicate a pre-segregation period where the origin has been replicated but the two copies have not yet committed to segregation. These origins bounce randomly, separating transiently and coming back together, before finally committing to segregate to opposite poles. This bouncing behaviour suggests that the process of segregation is separated in time from the process of replication of the chromosome. Furthermore, this observation suggests that *V. cholerae* chromosomes remain cohered for some time after replication before segregation occurs. Several lines of evidence indicate a period of cohesion of *E. coli* chromosomal loci after replication (Sunako *et al*., 2001; Bates and Kleckner, 2005).

### Origin behaviour is biphasic and correlated with progress through the cell cycle

**Fig. 5:**
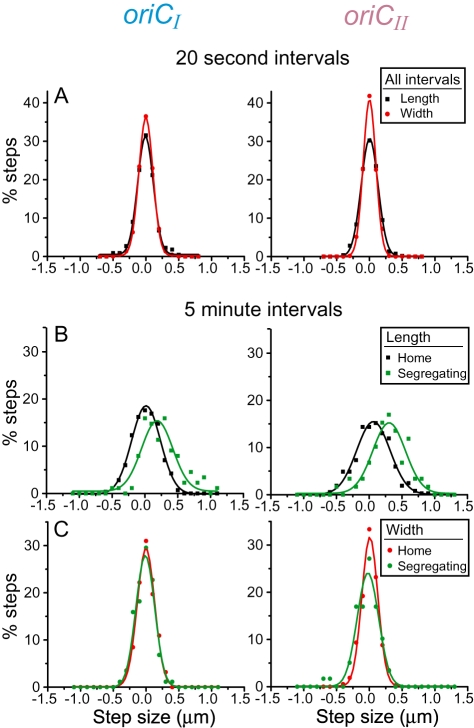
Movement of origins is greater in the length axis and is directionally biased in the length axis during the segregating phase. A. The distribution of 20 s step in the length (black squares) and width (red circles) axes fit Gaussian functions (black and red lines) centred around zero, as expected for random motion. The graphs represent 222 and 526 steps for *oriC*_*I*_ and *oriC*_*II*_ respectively. B and C. The steps over 5 min intervals were analysed separately for the two different phases of motion and the two axes of the cell. In the home phase, steps fit Gaussian distributions centred around zero (black and red indicate length and width axes respectively). In the segregating phase (green), the centre of the step size distribution is shifted from zero in the length (B), but not width (C), axis indicating directional bias in the length axis. *oriC*_*I*_ distributions represent 284 and 88 steps in the home and segregating phases respectively. *oriC*_*II*_ distributions represent 369 and 59 steps in the home and segregating phases respectively. In both (A) and (B and C) the standard deviation is greater in the length axis than in the width axis, indicating larger steps in the long axis of the cell (see text).

Following origin motions over time in individual cells, we observed and characterized two distinct modes of behaviour of *V. cholerae* origins corresponding to periods of active segregation and periods between segregation events. Segregation begins when one focus separates into two distinct foci and includes directional origin movement across the cell. The examples of origin behaviour in individual cells in Fig. 3A–D demonstrate that between segregation events, the position of the origin is not fixed but is confined to a region, within which it exhibits rapid but apparently random motion (analysed in more detail below, Figs 5 and 6). The region of origin confinement is near the pole for *oriC*_*I*_ and near the mid-cell for *oriC*_*II*_ (Fig. 3A–H). We term this confined region the ‘home’ position. Comparison of the dynamic behaviour patterns from all cells (Fig. 3E and F) revealed that in every track, the position of the chromosome origin is variable in the home position, suggesting that the variation in the actual position of the chromosome origins within a cell population is largely due to variation over time within individual cells, rather than strictly to cell-to-cell variation. These two phases of chromosomal behaviour have been observed in *E. coli*, *Bacillus subtilis* and *C. crescentus* (Gordon *et al*., 1997; 2004; Webb *et al*., 1998; Viollier *et al*., 2004) with an emphasis on the segregating phase. We went on to quantitatively characterize movement in both phases to understand the dynamics of chromosomes throughout the cell cycle.

### The home position of each origin is differentially maintained

Given that chromosomal origins are not randomly distributed throughout the cell, and that *oriC*_*I*_ and *oriC*_*II*_ have unique distribution patterns, we asked how the origin’s home position is determined. We examined two possible scenarios. The first possibility is that the origins are consistently positioned at a particular actual distance from a pole. In this case, origin distances from the pole will be conserved regardless of the length of the cell. The slope of a linear fit of the home position versus cell length will be zero, and the intercept will indicate the distance from the pole (Fig. 2B, i). Fractional positions will be inversely proportional to cell length, and as a result, will fit a power function (*y* = a*x*^b^) where the exponent b = −1, and the scaling factor, a, indicates the fractional distance from the pole (Fig. 2B, ii). The second possible scenario is that the origin is localized to a relative position in the cell. The MinCDE system used by *E. coli* to identify the middle of the cell, regardless of length exemplifies this scenario (reviewed by Margolin, 2001). If the origins localize to a relative position in the cell, the slope from a linear fit of origin home positions versus cell length will reveal the relative position measured by the cell and the intercept should be near zero (Fig. 2B, iii). Conversely, a regression line from fractional positions versus cell length will have a slope of zero, and the intercept will indicate the relative position measured by the cell (Fig. 2B, iv). We found that different methods are used to maintain the home positions of *oriC*_*I*_ and *oriC*_*II*_.

**Fig. 4:**
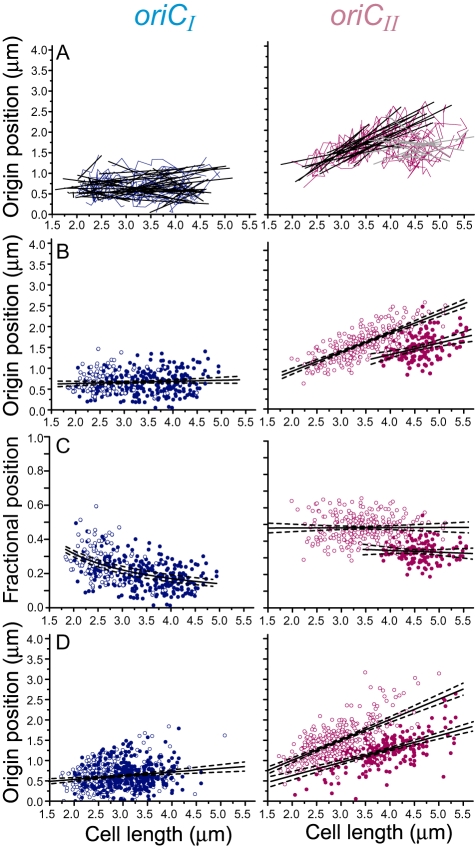
Between segregation events, *oriC*_*I*_ is positioned at a constant absolute distance from the pole and *oriC*_*II*_ is positioned at a constant relative distance from the pole. For analysis of home positioning method, origin tracks from 5 min interval movies were analysed. All *oriC*_*I*_ tracks as well as duplicated and segregated *oriC*_*II*_ tracks are plotted with the nearest pole at zero. This enables comparison of ‘home’ domains on opposite sides of the cell. Before an observed cell division, the poles are indistinguishable. For measurement of *oriC*_*II*_ position, using the nearest pole as zero gives a non-normal distribution of positions (not shown) indicating a bias in measurement. Thus, before observed divisions, single *oriC*_*II*_ tracks are plotted with an arbitrary pole at zero; after cell division, the new pole was used as the zero. A. Regression lines for individual origins in the home position (black lines) correspond to the home position tracks as shone in Fig. 3E and F (thin blue or pink lines). The average slope for *oriC*_*I*_ is 0.07. The average slope for *oriC*_*II*_ is 0.48 when single copy and 0.15 when duplicated. B. Regression analysis of the actual positions of the time-lapse foci taken together as a whole. *oriC*_*I*_ fits a line with a slope near zero (*y* = 0.03*x* + 0.58) and *oriC*_*II*_ fits a line with a slope near 0.5 (*y* = 0.49*x* − 0.02) when single copy and near 0.25 (*y* = 0.29*x* + 0.21) when segregated. C. The fractional positions of the time-lapse origin versus cell length. *oriC*_*I*_ fits a line with the equation *y* = 0.58*x*^(−0.96)^ and *oriC*_*II*_ fits a line with a slope of zero (*y* = 0.001*x* + 0.48) and (*y* = −0.01*x* + 0.39) for single and double spots respectively. D. Regression analysis for a population of still images of ∼500 cells yields lines of (*y* = 0.09*x* + 0.35) for *oriC*_*I*_ and (*y* = 0.50*x* + 0.006) and (*y* = 0.33*x* − 0.03) for single and double copies of *oriC*_*II*_ respectively. These fits are comparable to those in (B) for the origins followed by time-lapse microscopy and confirm that *oriC*_*I*_ is positioned at an actual distance from the pole regardless of the number of origins in the cell and *oriC*_*II*_ is positioned at a relative position in the cell. Solid lines indicate the fit of the data. Dotted lines represent the 5 and 95% confidence intervals of the fit calculated by Microcal Origin 6.0 (Microcal Software, Northampton, MA). A single origin in a cell is indicated by an open circle. Once segregated, origins are indicated by filled circles.

All analyses indicate that *oriC*_*I*_ is positioned by a mechanism that measures actual distances (Fig. 4). *oriC*_*I*_ home positions are maintained through cell division and as a result of asymmetric segregation do not depend on the number of segregated origins in the cell. Therefore, *oriC*_*I*_ positioning was evaluated without regard to the number of origin foci in the cell. First, linear regression of positions of individual *oriC*_*I*_ tracks in the home phase yields a median slope near zero (0.07) (Fig. 4A, Table 1). Furthermore, linear regression of all home *oriC*_*I*_ together also gives a slope near zero (0.03) and an intercept of 0.58 µm (Fig. 4B). Fractional positions fit a power function with an exponent that approaches −1 (−0.96) and a scaling factor of 0.58 (Fig. 4C). Together these data indicate that *oriC*_*I*_ is positioned at a constant actual distance (average ∼0.6 µm) from the pole independent of cell length or cell cycle phase.

**Table 1:** Rates of change in 5 min time-lapse.

*oriC*	Phase	Δ spot position/time (µm min^−1^)*	Δ cell length/time (µm min^−1^)*	Δ spot position/Δ cell length*	♯ track segments	Mean points/ track
I	Home	0.002 ± 0.006	0.025 ± 0.008	0.07 ± 0.28	26	11.4
	Segregating	0.043 ± 0.014	0.022 ± 0.009	2.09 ± 0.81	12	8.1
II	Home	0.009 ± 0.010	0.018 ± 0.007	0.48 ± 0.46	23	12.2
	Segregating	0.061 ± 0.028	0.020 ± 0.015	2.39 ± 1.23	22	3.7

* Mean ± SD.

In contrast, similar analyses of *oriC*_*II*_ in its home position indicate that it is localized by a mechanism that measures relative position in the cell (Fig. 4). Because the distributions for cells with one or two discrete *oriC*_*II*_ foci are different, they were separated for this analysis. Analysis of the actual positions of individual tracks for single *oriC*_*II*_ foci versus cell length resulted in a median slope of 0.48 (Fig. 4A, Table 1). Linear regression of the actual positions of all single *oriC*_*II*_ foci tracks together yielded a slope of 0.49 and a near zero intercept of −0.02 µm (Fig. 4B). Conversely, linear regression of fractional positions resulted in near zero slopes of 0.001 and −0.011 and intercepts of 0.48 and 0.39 for cells with single and double *oriC*_*II*_ foci respectively (Fig. 4C). These analyses all support a relative positioning mechanism where *oriC*_*II*_ tracks the mid-cell when present as a single focus and approaches the nascent mid-cells of the future daughter cells when segregated.

To confirm that this result was a general property of the population rather than that of the relatively small number of cells followed by videomicroscopy (*n*_*oriCI*_ = 13, *n*_*oriCII*_ = 16), regression analysis was repeated with spot positions from static images of ∼500 cells for each chromosome (Fig. 4D). In static images it is impossible to determine if the origins are at home or segregating. Therefore, all *oriC*_*I*_ positions were compared with the nearest pole, and *oriC*_*II*_ positions were parsed by the number of spots in the cell. Single *oriC*_*II*_ foci were measured from an arbitrary pole while segregated *oriC*_*II*_ foci were compared with the nearest pole. Regression analysis with population data further supports the model that *oriC*_*I*_ is positioned at a constant actual distance from the pole while *oriC*_*II*_ is positioned at a constant relative distance from the pole (Fig. 4D).

### Origin motion is not equal along the two axes of the cell

To characterize the dynamic motion of origins throughout the cell cycle, we first analysed the changes in origin position in single 20 s or 5 min time-lapse intervals. In sequences of 20 s intervals, no initial segregation events were observed making it impossible to distinguish home and segregating phases. Therefore, all time intervals were considered together. The 5 min intervals were separated by phase.

For both origins, the motion was random in 20 s intervals and 5 min home phase intervals. The distributions of positional change fit Gaussian functions centred on zero (Fig. 5A–C, Table 2) demonstrating that steps are equally likely in either direction.

**Table 2:** Step size statistics.

			Step size (µm)*	
Interval	Phase	Axis	*oriC*_*I*_	*oriC*_*II*_
20 s		Length	−0.005 ± 0.15 (222)	0.001 ± 0.13 (526)
20 s		Width	0.001 ± 0.12 (222)	0.002 ± 0.09 (562)
5 min	Home	Length	0.012 ± 0.202 (284)	0.059 ± 0.260 (369)
5 min	Segregating	Length	0.247 ± 0.290 (88)	0.294 ± 0.283 (59)
5 min	Home	Width	−0.004 ± 0.137 (284)	0.004 ± 0.132 (369)
5 min	Segregating	Width	−0.020 ± 0.142 (88)	−0.042 ± 0.185 (59)

* Mean ± SD (*n*).

In addition, steps are larger in the length axis than in the width axis. For both chromosomes, the standard deviation of step size is greater in the length axis than in the width axis (Fig. 5A–C, Table 2), indicating that origins are more likely to move farther in the length axis than in the width axis in a given interval. For steps in 20 s intervals, the differences in standard deviation are small but statistically significant (*F*-test *P* = 0.003 and *P* << 0.0001 for *oriC*_*I*_ and *oriC*_*II*_ respectively). Bias towards larger steps in the long axis of the cell is more apparent at 5 min intervals indicating a continuous effect. Again, the standard deviation is greater in the length axis for both origins (*F*-test *P* << 0.0001 for both origins). Thus motion in 20 s intervals and in 5 min home phase intervals is random in direction, but biased in magnitude with longer steps in the length axis. This bias in movement indicates that motion is differentially confined in the two axes of the cell. Furthermore, *oriC*_*I*_ and *oriC*_*II*_ behaved similarly between 20 s and 5 min intervals in both the length and width axes (Fig. 5), demonstrating that both origins experience similar constraints.

### Motion in the segregating phase is directed

While motion in the home phase is random, motion in the segregating phase is directionally biased along the long axis. As noted above, steps are centred about zero in the home phase; that is, they are equally likely to occur towards or away from the nearest pole. In the segregating phase, steps in the length axis are not centred about zero, but rather have average values of 0.25 ± 0.29 and 0.29 ± 0.28 µm for *oriC*_*I*_ and *oriC*_*II*_ respectively (Fig. 5B, Table 2). Thus these steps are both longer and directionally biased. This means that in the segregating phase, *oriC*_*I*_ is more likely to move towards the new pole than the old pole, and *oriC*_*II*_ is more likely to move away from the mid-cell. In the width axis, differences in step sizes between the movement phases are not statistically significant (Fig. 5C) indicating that motion perpendicular to the long axis of the cell is not affected by the directional segregation of the origins. This directional bias in motion during segregation indicates that segregation of *V. cholerae* chromosome origins does not occur by random motion, but rather through a directed process operating strictly along the long axis of the cell. Interestingly, the mobility of the origins is clearly not more constrained during segregation than it is during the phases of the cell cycle between segregation events. Thus, the directed process driving segregation must operate while superimposed on the fairly rapid, random motions that the origins undergo while confined in the home positions, without measurably suppressing these random motions, and without apparently altering the local constraints experienced by the chromosomal segments.

### Cell growth contributes to the motion of segregating origins

As discussed above, the home position of *oriC*_*I*_ is not influenced by cell length. Conversely, the home position of *oriC*_*II*_ moves from the pole at about half the rate of cell growth, consistent with this origin maintaining a position near the mid-cell (Table 1). However, during segregation, *oriC*_*I*_ moves about two times faster than cell growth (0.04 ± 0.01 versus 0.02 ± 0.01 µm min^−1^), and *oriC*_*II*_ moves about three times faster than the rate of cell growth (0.06 ± 0.03 versus 0.02 ± 0.02 µm min^−1^) (Table 1). In addition to the directional bias in motion described above, this difference in average rates between origin movement and cell growth indicates a non-diffusive, directed segregation mechanism.

Nevertheless, we found that cell growth does contribute to the segregation of the origins. We asked how much of the motion could be accounted for by cell growth alone. While cell wall synthesis is a prominent feature of cell growth, inhibition of cell wall synthesis at the septum in *E. coli* (Gordon *et al*., 1997) or along the body of the cell in *B. subtilis* (Webb *et al*., 1998) does not affect chromosome segregation. Membrane growth and increases in bulk cytosol are also important features of cell growth. The dynamics of the membranes and cytoplasm growth are not well understood. Assuming that incorporation of new material to the cell is evenly distributed along the body of the cell, we calculated the expected change in position if cell growth was the only factor influencing origin movement. The ratio of the expected difference in position from cell growth to the total difference in position indicates the percentage of motion that can be accounted for by cell growth. We found that during segregation, the median contribution of cell growth to origin movement was 19%[with an intraquartile (25–75 percentile) range of 2.4–44%] for *oriC*_*I*_ and 16% (with an intraquartile range of 2.8–32%) for *oriC*_*II*_. Thus cell growth can account for a substantial portion of the movement of the origins, but cannot account for all of the motion observed.

### Origins in the home phase move subdiffusively in similarly sized caged domains

**Fig. 6:**
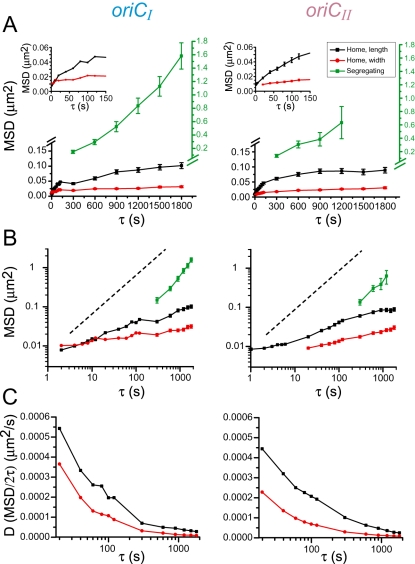
Origins behave subdiffusively and are caged in both axes by 10 min. MSD for time intervals (τ) between 1 and 1500 s are plotted on liner axes (A) and logarithmic axes (B) for both *oriC*_*I*_ and *oriC*_*II*_. Displacement in the home phase approaches a horizontal asymptote in both the length (black) and width (red) axes indicating that motion is restricted to a caged domain. In the segregating phase, displacement along the length axis (green squares) is dramatically greater than any movements in the home phase. In (A), note the change in vertical axes. Insets in (A) show expansions of the short-time intervals. In (B), the slopes of MSD versus τ are less than 1 in the home phase revealing subdiffusive behaviour of the origins (dashed line has slope of 1 for comparison). When motion is subdiffusive, the apparent diffusion coefficient is dependent on τ. The apparent diffusion coefficient (MSD/2τ) is plotted as a function of τ for both axes (C). Displacement of *oriC*_*I*_ is measured with pole as the frame of reference in the length axis. Displacement of *oriC*_*II*_ is measured with the mid-cell as the frame of reference. Error bars represent standard error of the means. These figures represent analysis of 16, 17 and 32 *oriC*_*I*_ tracked at 2 s, 20 s and 5 min intervals respectively, and 17, 29 and 28 *oriC*_*II*_ tracked at 1 s, 20 s and 5 min intervals respectively.

To quantitatively characterize how the origin position evolves through time, and to distinguish between diffusive, subdiffusive (caged) and superdiffusive (directed) motion, we extended our analysis to look at the change in position between intervals greater than one frame in sequences collected at 1 or 2 s, 20 s and 5 min intervals. The mean squared displacement (MSD) of origin position is plotted against the time interval (τ) (Figs 6A and B). In the home position, MSD in both axes approaches a horizontal asymptote indicating that movement is restricted to a caged domain (Fig. 6A). However, in the segregating phase, neither origins appears caged (Fig. 6A).

While the diameter of the caged domain (two times the square root of the horizontal asymptote) is different in the length and width axes, the origins of both chromosomes have comparable domains of movement. For both origins, the caging diameter in the width axis is similar (∼0.4 µm) and smaller than the width of the cell (∼0.75 µm). Likewise, the caging diameter in the length axis (∼0.6 µm) is only a fraction of the cell length (2.0–5.5 µm). Notably, the diameter of the caged domain in the length axis (∼0.6 µm) approaches the width of the cell (∼0.75 µm). Thus, because of the physical restrictions of the cell, the range of motion is more confined in the width axis and this is reflected in the smaller caging radius in this axis. The similarities in cage dimensions for both origins imply that both experience similar microenvironments, though at different locations in the cell.

The slope of MSD versus time plotted on a log scale indicates if random diffusion is governing the movement of the particle (slope = 1), if the movement is less than that expected by diffusion and thus is constrained (slope < 1), or if the motion is directionally biased or otherwise superdiffusive (slope > 1). In the home position, both origins behave subdiffusively on all time scales observed (Fig. 6B). This is likely a consequence of the fact that we are observing the motion of one position in a long polymeric chain. The connection to the rest of the chromosome will limit the range of movement for any particular locus. In the segregating phase, directed motion (evidenced by the bias in step direction) is superimposed on the subdiffusive behaviour of the origins making the slope of MSD versus time difficult to interpret. Indeed, the MSD analysis reveals that origin behaviour is clearly different in the two phases (Fig. 6A and B), indicating that different kinds of forces govern origin mobility in the home and segregating phases. Together the MSD analyses and the bias in step direction suggest that motion during segregation is not superdiffusive but rather a biased random walk.

When a particle is behaving diffusively, the diffusion coefficient (D) can be derived from the slope of MSD versus time plotted on a linear scale. In our case, however, the origins are behaving subdiffusively so D represents an apparent diffusion coefficient, which is dependent on the time interval of measurement. When displacement is measured in a single dimension, D = MSD/2τ where τ is the time interval between measurements (Berg, 1993). The apparent diffusion coefficient for both origins in both cell axes was generated for each time interval and plotted versus the time interval (Fig. 6C). The apparent diffusion coefficient is comparable for the two origins, and at intervals greater than 10 s it is greater in the length axis than in the width axis. This analysis further supports the idea that motion of origins is differentially constrained in the length and width axes of the cell, but the fine-scale motions of the two chromosomal origins are similar to each other.

### Origin motion within individuals partially accounts for the variability in origin position observed in static populations

**Fig. 7:**
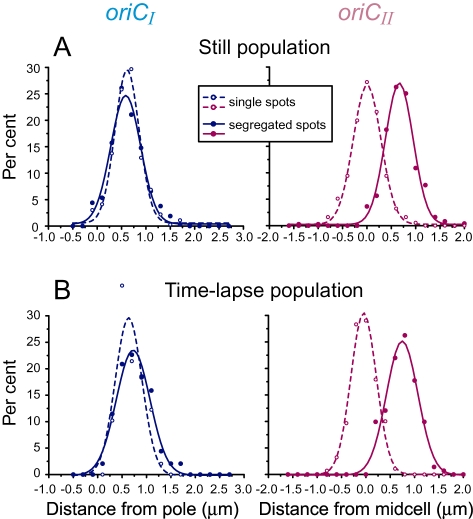
The population distribution of origin positions. Distributions of origin position from either populations of (A) 425 cells each imaged at a single point in time, or (B) the 5 min interval time-lapse data are plotted versus distance from the pole for *oriC*_*I*_ and distance from the mid-cell for *oriC*_*II*_. Cells with single origin spots were examined separately from those with two origin spots. For *oriC*_*I*_, the distribution is the same for single and double spots, while for *oriC*_*II*_ the relative positions clearly change after duplication and segregation. Curved lines show fits to a Gaussian function. The standard deviation for all curves is 0.3 µm.

To assess if motion observed at the home position in these individual cells reflects the range of positions observed in still images for larger populations of cells, we compared the calculated caging radii for individuals observed by time-lapse to the range of origin positions in a large number of static images (Fig. 7). Actual distances between origins and the nearest pole or the mid-cell were measured for a population of 496 cells for *oriC*_*I*_ and 523 cells for *oriC*_*II*_. The distributions of origin positions measured along the length axis among the population in these static images of cells are represented by standard deviations of 0.3 µm for all *oriC*_*I*_ foci measured from their closest pole and 0.3 µm for *oriC*_*II*_ foci measured from the mid-cell. If each position in the 5 min time-lapse datasets is taken as an independent measurement, the standard deviation for both origins is also 0.3 µm indicating that the time-lapse dataset adequately represents the larger population. However, assuming no variation in the position of the cage among individuals, the expected standard deviation for caged objects is equal to the caging radius/√2. As the caging radius we observe in the length axis by analysing the plots of MSD versus τ is 0.3 µm, the expected deviation for positions in the length axis would be 0.21 µm if the variation in the population were due to motion alone. Thus, the variation in the population reflects primarily the intrinsic origin mobility, but there is also some contribution from cell-to-cell variation.

## Discussion

**Fig. 8:**
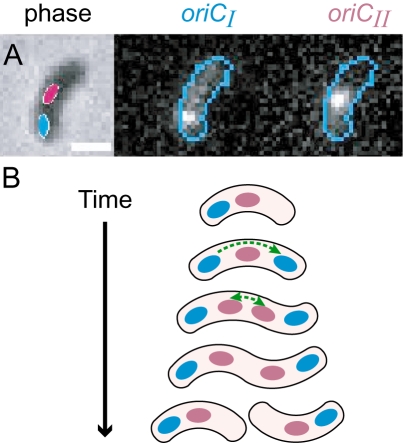
Relative size and temporal positioning of caged origin domains through the cell cycle. The scaled sizes of the ellipsoidal caging domains are shown on (A) an example cell and (B) a model cell through a cell cycle. The caged domains for *oriC*_*I*_ (blue) maintain a constant actual distance from the pole and the caged domains for *oriC*_*II*_ (red) maintain a constant relative position in the cell. Green arrows indicate directed movement during the segregating phase, which is asymmetric for *oriC*_*I*_ and symmetric for *oriC*_*II*_.

Here we report detailed time-lapse analysis of the gross and fine-scale dynamics of *V. cholerae* chromosome origins throughout the cell cycle, both of which are summarized in Fig. 8. Our analysis of fine-scale origin movements revealed (i) between segregation events, origins are not fixed in place but rather move subdiffusively within caged domains (ovals in Fig. 8) and (ii) during segregation, both origins move at comparable rates with directed motion superimposed on the rapid random subdiffusive motion characteristic of origin behaviour during maintenance at the home position. Several features of the caged domains are unexpected. First, the domains of each origin are of similar dimensions suggesting that the constraints and microenvironment experienced by each are similar, despite the dramatic differences in size, location and gross-scale behaviour of the two chromosomes. Second, motion is unequal in the two axes of the cell. It is not clear what leads to this phenomenon, but caging effects of the cell edges likely limit motion in the width axis of the cell. The gross origin behaviours we observed support those described by Fogel and Waldor (2005). Specifically, *oriC*_*I*_ is found near, but not at, the old pole (Fig. 8, blue ovals) and segregates asymmetrically from that pole early in the cell cycle (Fig. 8B, green arrow). *oriC*_*II*_ is found near the mid-cell before segregation (Fig. 8, red ovals) and segregates symmetrically to the quarter positions later in the cell cycle (Fig. 8B, double headed green arrow).

### How are chromosome origins positioned in the *V. cholerae* cell?

In bacteria, the poles are physically distinct from the rest of the cell providing a framework that enables the accumulation or exclusion of particles at or away from the pole (reviewed by Shapiro *et al*., 2002). In addition, in some rod-shaped bacteria, the MinCDE system facilitates the identification of the mid-cell (reviewed by Margolin, 2001). But how are components targeted to and maintained at positions that are neither the pole nor the mid-cell? What is the mechanism for measuring and enforcing the position of non-randomly distributed particles? At least two frames of measurement are theoretically possible; particles could be targeted positions that are fixed actual distances from distinct cell features, or alternatively they could be targeted to positions at fractional distances from a cell feature. Evidence presented here indicates that in *V. cholerae*, both frames of measurement are used to position the origins of the two chromosomes.

In *V. cholerae*, *oriC*_*I*_ is neither localized to a pole or to the mid-cell, but rather is found in a defined domain near the pole (Fig. 8). The position of the domain is insensitive to the length of the cell indicating that the ruler which positions *oriC*_*I*_ measures actual (as opposed to fractional) distances. How actual distances are measured in the cell, however, is not clear. Because the *oriC*_*I*_ domain is a constant distance from the pole, it seems likely that the pole is somehow involved in positioning this origin. One possibility is that the chromosome is excluded from the pole in a manner independent of cell cycle and that *oriC*_*I*_, the most distal portion of the nucleoid mass (Fogel and Waldor, 2005), is segregated to the most polar portion of the cell before it is physically excluded. It is not clear what would prevent the chromosome from occupying the polar cap region. Accumulation of ribosomes (Lewis *et al*., 2000; Mascarenhas *et al*., 2001) or other proteins at the poles could exclude *oriC*_*I*_ from the most polar regions of the cell, although it seems surprising that the amount of these components should not increase as the cell grows. Another possibility is that a measuring protein of a specific length indicates the position of the *oriC*_*I*_ domain from the pole. Such a protein anchored at the pole could enable the cell to physically measure the position of *oriC*_*I*_. Similar distance measuring proteins are used by bacteriophages to determine tail length (Abuladze *et al*., 1994; Vianelli *et al*., 2000) and by skeletal muscle cells to measure precise lengths in the sarcomere (Wang, 1996). However, because the distance between *oriC*_*I*_ and the pole is not precise and varies through time, we favour the hypothesis that this origin is simply excluded from the most distal region of the cell.

Simultaneously, *oriC*_*II*_ is positioned in the vicinity of the mid-cell regardless of the length of the cell (Fig. 8). In *E. coli* and *B. subtilis*, the FtsZ ring is positioned at the mid-cell by the MinCDE system, and a host of other proteins then form a complex upon the FtsZ ring to prepare the cell for division (reviewed by Margolin, 2001; Errington *et al*., 2003). Both FtsZ and MinCDE are found in the *V. cholerae* genome (Heidelberg *et al*., 2000), thus one possibility is that the cell directly or indirectly positions *oriC*_*II*_ using components of either the FtsZ ring or of the Min system. In *E. coli*, the positioning of FtsZ to the mid-cell is strikingly accurate. While, to our knowledge, FtsZ has not been localized in *V. cholerae*, it should be noted that *oriC*_*II*_ is not always positioned at precisely the mid-cell. In some cells it tracks the mid-cell closely, but in others it tracks a position slightly closer to the pole (around 40 or 60% of the cell length) and still in others *oriC*_*II*_ moves between positions at 40–50% of the cell length. The lack of precision may reflect an imprecise readout of the Min system. Alternatively, it could indicate that the positioning mechanism is independent of the Min-established mid-cell complexes. In addition, the variation in position may reflect that the anchoring point of chromosome II is not the origin.

Alternatively, the *parA* and *parB* partitioning genes on chromosome II could act to position *oriC*_*II*_ at the mid-cell. Interestingly, both *V. cholerae* chromosomes encode independent partitioning loci proximal to each origin of replication (Heidelberg *et al*., 2000). While the *par* genes encoded by chromosome I are more similar to other chromosomally encoded *par* genes, those from chromosome II are more similar to plasmid-encoded *par* genes (Gerdes *et al*., 2000; Heidelberg *et al*., 2000; Yamaichi and Niki, 2000). ParA from the *E. coli* plasmid pB171 oscillates from pole to pole independent of *minCDE* (Ebersbach and Gerdes, 2001). Moreover, this ParA positions pB171 at the mid-cell and plays a role in plasmid segregation (Ebersbach and Gerdes, 2004). Thus it is possible that the *par* loci on chromosome II position *oriC*_*II*_ at the mid-cell independent of the division plane machinery.

### What is the contribution of cell growth to origin segregation?

The classic hypothesis that cell wall growth provided the force to segregate chromosomes (Jacob *et al*., 1963) has been re-evaluated with the development of techniques for visualizing chromosome behaviour in live cells. Origin segregation in several species has been observed to be faster than cell growth (Glaser *et al*., 1997; Webb *et al*., 1998; Gordon *et al*., 2004; Viollier *et al*., 2004), indicating that bacteria must employ an active mechanism for chromosome segregation. In these studies, growth conditions were such that each cell contained one to two copies of its chromosome. While in these cases, an active segregation mechanism is hard to dispute, the quantitative contribution of cell growth (i.e. incorporation of new material throughout the cell) to origin segregation has largely been ignored. Recently, Elmore *et al*. (2005) tracked *E. coli* origins in fast-growing cells where two to four origin foci are present in each cell. These researchers did not observe a period of rapid and directed movement and determined that cell growth alone could account for the segregation of origin foci under these conditions. Several differences could account for the discrepancies between experimental systems. In fast-growth conditions when more origins copies are present, each would have a shorter distance to travel before establishing a new home. It is possible that in these conditions, the period of active movement is short enough that it is masked when looking for consecutive intervals of directed movement. Similarly, if the caged regions of the segregated origins overlap with the caged region of the parental origin focus, directed movement between parental and progeny domains may not be detectable. Alternatively, the cell may use different mechanisms to segregate chromosomes under different growth conditions. Cells may only need to rely on a directed mechanism under slower growth conditions.

Under our experimental conditions, *V. cholerae* origin segregation is only two to three times faster than cell growth and is slower (0.04 and 0.06 µm min^−1^ for *oriC*_*I*_ and *oriC*_*II*_ respectively) than the segregation observed in other species (0.1–0.3 µm min^−1^; Webb *et al*., 1998; Gordon *et al*., 2004; Viollier *et al*., 2004). We calculated that cell growth could account for nearly 20% of the motion observed during segregation of *V. cholerae* origins. Because origin segregation is more rapid in other species, cell growth likely makes a smaller contribution. While cell growth contributes substantially to origin segregation, it alone cannot account for all of the motion observed.

### How is the motion of DNA segments restricted within the cell?

By tracking the position of both origins through time, we observe that while each origin occupies a unique region of the cell, the position of neither origin is fixed. Motion in both axes is random in direction and both origins exhibit subdiffusive behaviour, indicating that the motion of these loci is confined. The subdiffusive behaviour may reflect the concentrated nature of the DNA in the cell and the constraints of packing a polymer that is three orders of magnitude longer than the length of the cell into the volume of the cell. If the diffusion coefficient, D, reflected free diffusion, it would correlate to the size of the molecule. In this case, the chromosomes differ in length by a factor of ∼3, yet the apparent diffusion coefficients are similar for both origins (Fig. 6C). However, if DNA is tethered at multiple discrete sites, then D is not dependent on the size of the whole polymer, but rather the length between tethering points (Marshall *et al*., 1997). The similar diffusion coefficients for both origins suggest that the density of connections between DNA and other parts of the cell (either directly to membranes or to transcription/translation complexes) is comparable for the two origin regions. Moreover, the apparent diffusion coefficients we observed in the long axis of the cell for *V. cholerae* origins between 20 and 100 s (1–4 × 10^−4^ µm^2^ s^−1^) are comparable to those observed for loci in yeast chromosomes and for a yeast cen plasmid calculated from similar time scales (5 × 10^−4^ and 3 × 10^−4^ µm^2^ s^−1^ respectively; Marshall *et al*., 1997). At longer time intervals (∼1–9 min), the apparent diffusion coefficient for the origin region of fast-growing *E. coli* chromosomes is 3–4 × 10^−5^ µm^2^ s^−1^ (Elmore *et al*., 2005), similar to the apparent diffusion coefficients we observed for *V. cholerae* on this time scale. Overall, these data indicate that prokaryotic and eukaryotic DNA experience similar constraints within the cell.

### How does movement of the origins reflect the domain structure of the *Vibrio* chromosomes?

Previous experiments using multiple different techniques suggest that there is large-scale domain organization in bacterial chromosomes. The organization of the domain surrounding the terminus of the chromosome is important for progression through the cell cycle (Lesterlin *et al*., 2005). The data presented here indicate that the origin regions of both *V. cholerae* chromosomes each occupy a physical domain that encompasses about 0.6 µm or 1/3 of the length of a newly divided cell (Fig. 8) and that the origin moves randomly within this domain. Analysis of chromosomal positions in fixed cells of other bacterial species also supports a large-scale domain for each locus that occupies roughly 1/3 of the cell (Niki *et al*., 2000; Viollier *et al*., 2004). Complementary with these positional observations, analysis of recombination frequencies between distant chromosomal positions indicates that the bacterial chromosome is organized into a small number of macro-domains between which recombination is limited (Valens *et al*., 2004). The size of the macro-domains predicted by recombination frequencies (∼1/4–1/6 of the chromosome) is on the same order as the range of movement observed during chromosome localization (∼1/3 of the length axis in small cells and 1/6 of the length in longer cells). Curiously, domains on opposite sides of the chromosome, which due to the circular nature of the chromosome presumably occupy similar regions along the length axis of the cell, do not recombine with each other (Valens *et al*., 2004). In the absence of a mechanism to distinguish opposite halves of the chromosome, this result implies spatial restriction in the width axis. The caging radii we observe in width axis predict domains that occupy about half of the cell width. This observation further supports the model that spatial restriction of the chromosome limits recombination between opposite halves of the chromosome. Together these results indicate large-scale organization of domains in bacterial chromosomes, though the functional consequences of these domains are not yet clear. Lastly, it is notable that the size of eukaryotic chromosome territories are 0.4–0.8 µm (Zink *et al*., 1998) which is very comparable to the domain dimensions reported here (0.4 µm by 0.6 µm). This again indicates that prokaryotic and eukaryotic chromosomes experience similar constraints.

## Experimental procedures

### Strain construction

Arrays of tandem copies of *lacO* or *tetO* sequences were integrated into the sequenced strain of *V. cholerae*, N16961 (provided by G. Schoolnik) (Heidelberg *et al*., 2000). For integration into *V. cholerae*, several modifications were made to pLau43 and pLau44 vectors carrying the *lacO* and *tetO* arrays (Lau *et al*., 2003). First, to generate vectors that could not autonomously replicate in *V. cholerae*, the pUC18 origins were replaced with the R6K origin, which requires the *pir* gene product for replication. The XhoI/BamHI fragment from pR6K (Epicentre, Madison, WI**)** was cloned into the large SalI/BamHI fragment from pLau43 and the XhoI/SalI fragment from pR6K was cloned into the large SalI/XhoI fragment from pLau44 to generate pAF104 and pAF105 respectively. These and subsequent vectors were propagated in EC100D pir+ (Epicentre, Madison, WI) or Pir2 (Invitrogen, Carlsbad, CA), two strains of *E. coli* which support R6K origins of replication. Second, the kanamycin-resistance gene in the *lacO* array was replaced with the chloramphenicol-resistance gene, *cat*. *cat* from pACYC184 (New England Biolabs, Beverly, MA) was PCR amplified and subcloned into pCR-Blunt II-TOPO (Invitrogen, Carlsbad, CA), excised with NsiI, and ligated into the NsiI sites in the KmR gene in pAF104 to generate pAF106. Third, an *oriT* was added to allow the array bearing vectors to be transferred to *V. cholerae* by conjugation. A SpeI fragment containing *oriT* from pHPV412 (provided by Patrick Viollier) was cloned into the SpeI site of pAF106 and pAF05 generating pAF119 and pAF118 respectively. Last, ∼1000 bp regions of genomic sequence from *V. cholerae* were added to these vectors to target integration to a specific locus. Chromosomal sequences were PCR amplified, subcloned into pCR-Blunt II-TOPO, removed with XbaI and SpeI, and ligated into the NheI site in either pAF119 or pAF118. Integration was targeted to regions between genes on opposite strands such that the arrays were integrated into overlapping terminator regions as opposed to promoter regions. Because these intergenic regions are small (usually < 50 bp), portions of the flanking genes were amplified together with the intergenic region to enlarge the homologous region and thereby increase integration efficiency. This strategy minimizes the chances of disrupting function or regulation of the genes near the integration site.

These constructs were then transferred by conjugation from the Pir2 *E. coli* strain into *V. cholerae* N16961 using the helper strains LS256 or LS980 (provided by Lucy Shapiro). Integrants were selected with 2 µg ml^−1^ chloramphenicol and/or 20 µg ml^−1^ gentamycin as appropriate. *E. coli* was counterselected with 100 µg ml^−1^ streptomycin. The *tetO* array was integrated between VCA1103 and VCA1104 (∼12 kb from the *oriC*_*II*_) in AVC89 and between VC2761 and VC2762 (∼13 kb from the *oriC*_*I*_) in AVC93. The *lacO* array was inserted between VC2761 and VC2762 (∼13 kb from the *oriC*_*I*_) in AVC89 and between VCA0010 and VCA0011 (∼9 kb from the *oriC*_*II*_) in AVC93. pLAU53 (Lau *et al*., 2003), which carries TetR-YFP and LacI-CFP under the control of the pBAD promoter, was electroporated into strains containing the arrays and selected with 100 µg ml^−1^ carbenicillin. AVC93 was used for time-lapse analysis of *oriC*_*I*_ and AVC89 was used for time-lapse analysis of *oriC*_*II*_.

### Growth conditions/sample preparation

Overnight cultures were inoculated from freshly plated freezer stocks in M9 glucose minimal media (Sambrook and Russell, 2001) supplemented with an additional 0.5% glucose, 0.01% casamino acids and appropriate antibiotics and grown on a roller at 37°C. Overnight cultures were diluted ∼1:100 in the fresh media and grown to early log (OD_600_ 0.2–0.3). A small aliquot (1–1.5 ml) was gently pelleted for 1 min at 3500 *g*. Cells were resuspended in fresh media without antibiotics. Arabinose was added to a final concentration of 0.2% to induce expression of TetR-YFP and LacI-CFP. Expression of the fluorescent proteins was induced for 30–45 min at room temperature without shaking. One microlitre of cells was then placed on a 1–2% agarose pad made with the same media for observation. The doubling time for cells on the microscope was 80–100 min. Expression of the fluorescent proteins from the pBAD promoter was leaky in these strains and fluorescent foci were frequently observed in the absence of arabinose.

### Microscopy

Cells were visualized on a Nikon Diaphot 300 inverted microscope at room temperature using a 60× objective. The image was further magnified by a 2× lens in front of the camera. YFP and CFP were visualized using filter set number 52017, which includes single-band exciters (Chroma, Rockingham, VT). Metamorph version 6.1 (Molecular Devices, Sunnyvale, CA) was used to drive the filter wheels and shutters. Images were collected on a cooled CCD camera (Princeton Instruments, Princeton, NJ). To understand the general localization patterns of each origin, images of a large numbers of still cells were acquired visualizing both YFP and CFP sequentially using exposure times of 0.4–2 s. For time-lapse analysis, we followed the origins tagged with the *tetO* arrays and visualized with TetR-YFP. Origins visualized with YFP were bright and easy to detect due to negligible background in early logarithmic phase. The CFP signal, on the other hand, is dimmer and harder to detect due to high background generated by autofluorescence of endogenous molecules in *V. cholerae*. Furthermore, frequent exposure to the CFP excitation light was phototoxic; in our experimental set-up, cells cease to grow with blue light exposures of 250 ms at intervals of 10 min or less. As we were interested in the dynamic behaviour on short-time scales, YFP was the superior fluorescent tag. Time-lapse sequences were acquired with fluorescence exposure times of 400 ms.

### Image analysis

#### Time-lapse studies

All image analysis was done with Matlab version 7.0 (The MathWorks, Natick, MA) using the image analysis toolbox. A Gaussian filter was applied to the raw fluorescence images and spots were detected by thresholding. Spot positions were calculated as the centroid of the thresholded region. The cell poles and centreline were determined manually for each cell in each frame of the time-lapse image sequences. The centrelines were determined with 6–10 linear segments. A line from the centroid of the fluorescent spot was drawn normal to the centreline. The distance between the spot centroid and the centreline indicated the position of the spot in the width axis. The distance between the normal line and the pole, along the centreline, indicated the position of the spot in the length axis (Fig. 2). To follow an origin through a time-lapse movie, spots were tracked by automatically associating each spot with the closest spot in the following frame in the XY plane. When spots divided, a new track was initiated and the parental spot was added to both daughter tracks.

#### Population studies

To identify cell bodies, phase images were thresholded. The identified regions were filtered according to size and width to eliminate touching cells. We then used an automated algorithm to identify the poles and the centrelines for each cell. First, the poles were identified as the two most distant points on the outline of the thresholded mask. Then, for each pixel along one side of the outline, the nearest pixel on the opposite side of the bacterium was identified, and the midpoint between them was calculated. The sum of the distance between the midpoints generates the length of the bacterium and the line drawn through the midpoints generates the centreline of the bacterium. Spots were identified and their positions were measured as in the time-lapse.

### Motion analysis

For quantitative analysis, the segregation phase begins with initial separation (i.e. the interval during which one focus separates into two distinct foci) and ends when the chromosome reaches a new home domain.

For *oriC*_*I*_, length measurements in the home position were always from the closest pole and length measurements in the travelling phase were always from the old pole. Thus when segregation was observed, both spots were measured from the old pole. As soon as the segregating spot established a new home position, its distance was measured from the new pole to enable comparisons with other home phase origins. For *oriC*_*II*_, at the beginning of a time-lapse sequence, an arbitrary pole was chosen as the reference point. During the segregation phase, the reference point was the pole from which the origin was moving away. Thus pairs of sister origins were measured from opposite poles during segregation. This allowed comparisons of all origins during segregation. When a new home is established, the closest pole became the reference point for regression analysis. After a cell division, the new pole was used as the reference point for *oriC*_*II*_.

To estimate the motion attributable to cell growth, we calculated the expected position if uniform growth along the body of the cell was responsible for the change in position using the following equation [distance_(*n*)_/cell length_(*n*)_] × cell length_(*n*+1)_ = expected distance_(*n*+1)_. Expected distance_(*n*+1)_/actual distance_(*n*+1)_ gives the proportion of motion that can be attributed to cell growth.

For MSD (Qian *et al*., 1991; Berg, 1993), we calculated the average change in origin position in overlapping sequential intervals of one to six frames from all time-lapse movies for each time interval, 1 or 2 s, 20 s and 5 min. For *oriC*_*I*_, differences in position were based on the nearest/home pole. For *oriC*_*II*_, differences in position in the length axis were measured from the mid-cell.

Extrapolation from the shortest time intervals indicates that MSD values do not go through the origin of the graph but rather cross the *y* axis at about 0.01 µm^2^ (Fig. 6, see inset). This gives an upper limit for the measurement noise, which is 0.1 µm or about 1 pixel in our experimental set-up. This estimate is consistent with the error associated with the process of measuring the positions of the spot in the cell frame of reference.

## Supplementary material

The following supplementary material is available for this article online.

**Fig. S1.** The distributions of origin positions are the same in reciprocally marked strains.

This material is available at part of the online article from http://www.blackwell-synergy.com
