# Supplementary material for: Fine-scale time-lapse analysis of the biphasic, dynamic behaviour of the two Vibrio cholerae chromosomes
Source: Mol Microbiol. 2006 Apr 21;60(5):1164–78. doi: 10.1111/j.1365-2958.2006.05175.x (PMC2779472; doi:10.1111/j.1365-2958.2006.05175.x)
Supplement: Supplementary file 1 [file mmi0060-1164-SD1.doc]

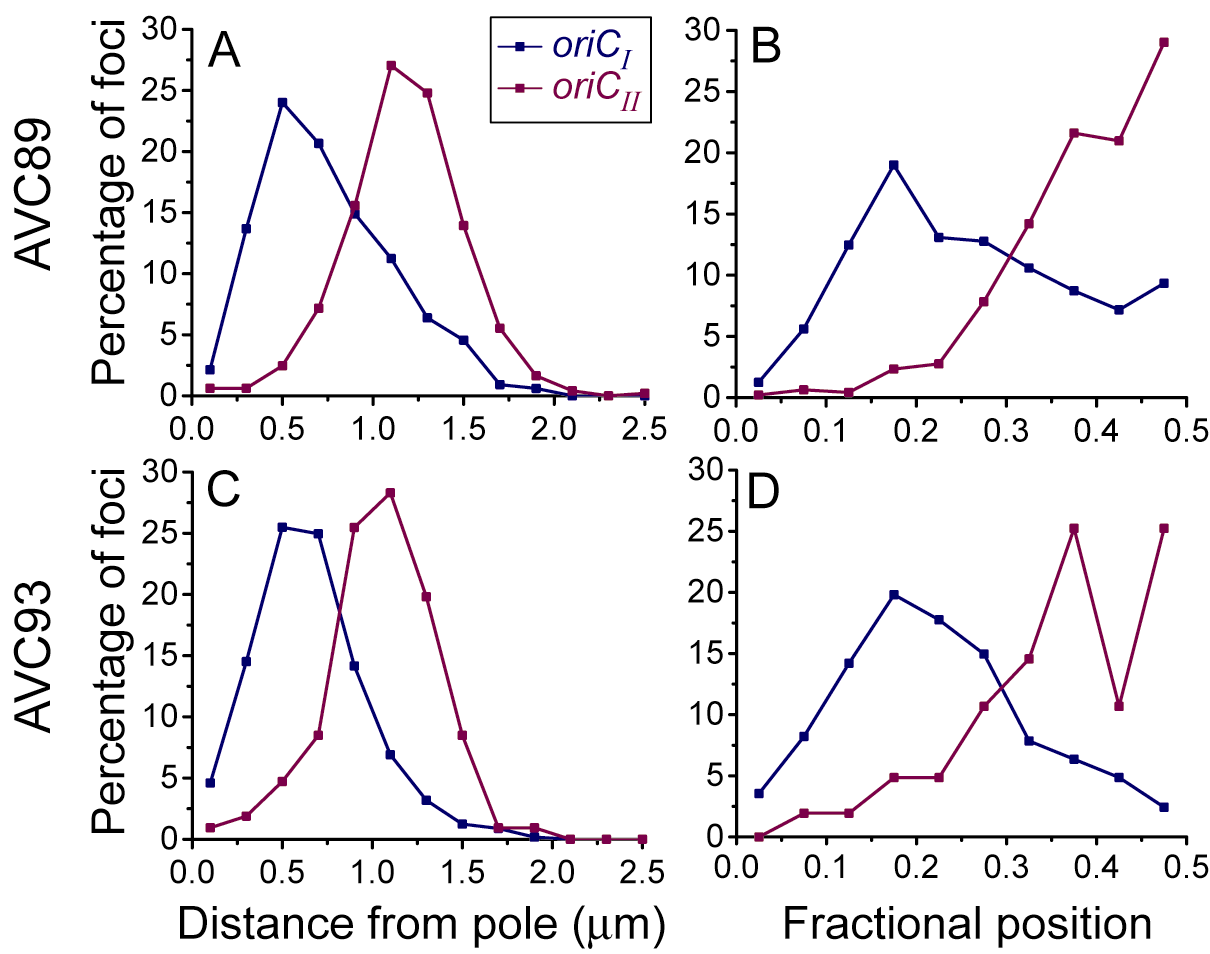


**Supplemental Figure: The distributions of origin positions are the same in reciprocally marked strains.** In AVC89 (A&B), the *lacO* array was inserted near *oriC_I_* and the *tetO* array was inserted near *oriC_II_*. In AVC93 (C&D), the *tetO* array was inserted near *oriC_I_* and the *lacO* array was inserted near *oriC_II_*. The actual (A&C) and fractional (B&D) positions of *oriC_I_* and *oriC_II_* were measured in 425 cells from each strain.
